# Meta-analysis of gene coexpression networks in the post-mortem prefrontal cortex of patients with schizophrenia and unaffected controls

**DOI:** 10.1186/1471-2202-14-105

**Published:** 2013-09-26

**Authors:** Meeta Mistry, Jesse Gillis, Paul Pavlidis

**Affiliations:** 1 Canadian Institute of Health Research/Michael Smith Foundation for Health Research (CIHR/MSFHR) Graduate Program in Bioinformatics, University of British Columbia, Vancouver, BC Canada; 2 Stanley Institute for Cognitive Genomics, Cold Spring Harbor Laboratory, One Bungtown Road, NY 11724, USA; 3 Department of Psychiatry, University of British Columbia, Vancouver BC, Canada; 4 Centre for High-throughput Biology, 177 Michael Smith Laboratories, 2185 East Mall, University of British Columbia, Vancouver BC, Canada

**Keywords:** Schizophrenia, Microarray, Gene coexpression network, Postmortem brain

## Abstract

**Background:**

Gene expression profiling of the postmortem human brain is part of the effort to understand the neuropathological underpinnings of schizophrenia. Existing microarray studies have identified a large number of genes as candidates, but efforts to generate an integrated view of molecular and cellular changes underlying the illness are few. Here, we have applied a novel approach to combining coexpression data across seven postmortem human brain studies of schizophrenia.

**Results:**

We generated separate coexpression networks for the control and schizophrenia prefrontal cortex and found that differences in global network properties were small. We analyzed gene coexpression relationships of previously identified differentially expressed ‘schizophrenia genes’. Evaluation of network properties revealed differences for the up- and down-regulated ‘schizophrenia genes’, with clustering coefficient displaying particularly interesting trends. We identified modules of coexpressed genes in each network and characterized them according to disease association and cell type specificity. Functional enrichment analysis of modules in each network revealed that genes with altered expression in schizophrenia associate with modules representing biological processes such as oxidative phosphorylation, myelination, synaptic transmission and immune function. Although a immune-function enriched module was found in both networks, many of the genes in the modules were different. Specifically, a decrease in clustering of immune activation genes in the schizophrenia network was coupled with the loss of various astrocyte marker genes and the schizophrenia candidate genes.

**Conclusion:**

Our novel network-based approach for evaluating gene coexpression provides results that converge with existing evidence from genetic and genomic studies to support an immunological link to the pathophysiology of schizophrenia.

## Background

Schizophrenia is a severe psychiatric disorder with an elusive etiology. Gene expression profiling of the postmortem human brain has been frequently used as a means to investigate patterns of molecular disruption in the brains of patients with schizophrenia. One of the most common types of analysis applied to expression profiling data is differential expression; which is used to identify over- or under-expressed genes associated with the illness. Candidate genes identified from expression profiling studies in schizophrenia have implicated alterations in different cellular systems, including myelination, synaptic transmission, metabolism, and ubiquitination [1]. These findings are not always replicated across studies, nor have they been successfully integrated into a comprehensive biological framework.

In our previous work, we used a large combined cohort to identify a meta-signature of genes which are consistently differentially expressed in the prefrontal cortex of patients with schizophrenia [2]. The functions reflected in these genes are diverse and the interactions among them are largely unexplored. Because gene function is partly defined by interactions with other genes (at the biochemical, physical interaction, genetic or regulatory levels), it is attractive to apply gene coexpression network analysis to aid in interpretation.

In general, gene networks can be analyzed to identify higher-level features of gene-gene relationships based on graph theoretic considerations such as node degree or clustering coefficient [3-5]. Evaluating the broader network structure allows us to detect modularity in the graph, or groups of densely connected nodes with sparse connections between groups. Characterization of these ‘modules’ can convey useful information as they may be associated with specific molecular complexes or functions, yielding hypotheses that would be difficult to ascertain based on a gene-by-gene analysis. It is important to note that the terminology “gene coexpression network” refers to a sparse representation of the correlation structure among genes, and that such networks are not amenable to straightforward interpretation in the way that protein interaction or metabolic networks are. However, a key advantage of coexpression is that there is relatively abundant data, so “condition-specific networks” can be constructed. Thus one can evaluate differences between condition-specific networks to help elucidate systems level molecular dysfunction.

Such coexpression network analyses have recently been applied to a number of postmortem human brain expression profiling datasets for examining general transcriptome patterns of the CNS [6], and to interrogate the molecular basis of neuropsychiatric disease [7-10]. Torkamani and colleagues [8] conducted a network analysis by combining two independent schizophrenia expression profiling datasets. Expression data was merged across control and schizophrenia cohorts and modules of coexpressed genes were characterized according to disease characterization, cell type specificity and the effects of aging. A more recent cross-cortical network study was carried out by Roussos et al. [10] using control and schizophrenia samples across four different brain regions. Discrete modules of coexpressed genes displayed high preservation between control and schizophrenia networks for all but one module. Brain regional differences were assessed with an analysis of variance comparison of module eigengene expression, with changes only observed in the control network. Chen et al. [11] also explored networks using combined data from schizophrenics and controls. Two modules were associated with genes differentially expressed with disease across the datasets; one which was specific to cerebellar cortex and the other identified across all brain regions. They did not report any differences in networks between schizophrenics and controls. Although Chen et al. used four data sets, they were not independent as three of the datasets used samples from the same brain collection.

In this study, we applied coexpression network analysis to seven independent gene expression studies of the prefrontal cortex to demonstrate, in agreement with previous studies, an overall similarity in transcriptome organization between healthy controls and individuals with schizophrenia. We then examined network properties of genes we previously reported to be differentially expressed in schizophrenia [2] within each network to reveal features of these genes that are not observed with other functional gene groups or other brain-related disease gene sets. Finally, using a network clustering approach, we found evidence for functional dysregulation of immune-related processes in schizophrenia. Our results complement previous gene expression and genetics evidence supporting an immunological aspect of the disorder.

## Results

**Figure 1:**
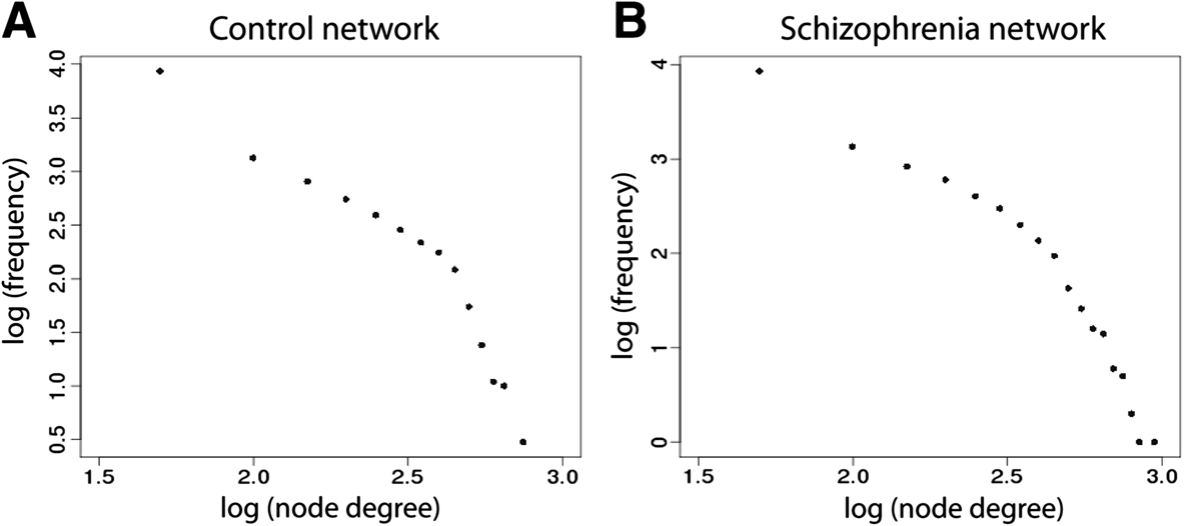
**Connectivity distribution of control and schizophrenia networks.** The control brain network **(A)** and the schizophrenia brain network **(B)** connectivity distribution on a log10-log10 scale. Plotted on the x-axis is the number of links versus the number of genes that have the corresponding number of links on the y-axis.

We constructed two gene coexpression networks; one representing the control human prefrontal cortex and the other representing the prefrontal cortex in schizophrenia (referred to as CTL and SZ, respectively). The schizophrenia and control groups had no significant differences in age and PMI (Table 1). Sex differences were assessed by use of a chi-squared test for equality of proportion, and we observed no significant difference (p = 0.1). Brain pH was significantly different (*t*-test; p = 0.001). Each network was comprised of 12,582 genes (nodes), and 392,606 coexpression ‘links’ among them. The two networks had similar values in the average clustering coefficient (p > 0.1), but average shortest path length across nodes differed slightly (p < 0.01). These network properties are summarized in Table 2. Both networks exhibited a ‘heavy-tailed’ node degree distribution, with most of the genes interacting with few partners and a small proportion of genes displaying ‘hub’-like behaviour interacting with many genes. In the literature, such distributions are sometimes described as ‘scale free’. We used a linear regression of the log-scale node degree distribution to examine this in our networks (Figure 1). While a linear fit explains over 80% of the variance in node degree distribution, (CTL R^2^ = 0.857; SZ R^2^ = 0.872), the fit is not good at the extremes. Based on more stringent criteria (which we endorse), our networks are not ‘scale-free’ [12]. However, the ‘heavy-tailed’ nature of the node degree distributions in our networks is typical of other ‘biologically relevant’ networks cited in the literature [13,14].

**Table 1:** Summary of demographic variables across combined cohort

	**Control**	**Schizophrenia**	**P-value**
Number of Subjects	153	153	
Age	56.25 ± 20	55.27 ± 19	p = 0.67
Sex	101 M : 52 F	113 M : 40 F	p = 0.1
Brain pH	6.5 ± 0.28	6.39 ± 0.29	p = 0.001
PMI	21.95 ± 15.3	22.65 ± 15.2	p = 0.69

F, female; M, male; PMI, post-mortem interval. There were 319 samples collected across seven datasets of which 306 passed quality control analysis. The summary demographics (mean ± standard deviation) and *t-* test p-values for group differences are shown for those subjects used in the analysis. For sex we report the p-value generated from a chi-squared test for equality of proportions.

**Table 2:** Whole network properties of the control and schizophrenia brain networks

	**Control**	**Schizophrenia**
Non-connected nodes	2356	2288
Maximum node degree	747	935
Mean node degree	77	76
Shortest path length	3.34	3.32
Cluster coefficient	0.29	0.29
log-log fit (R^2^)	0.857	0.872
Number of modules	25	25

**Figure 2:**
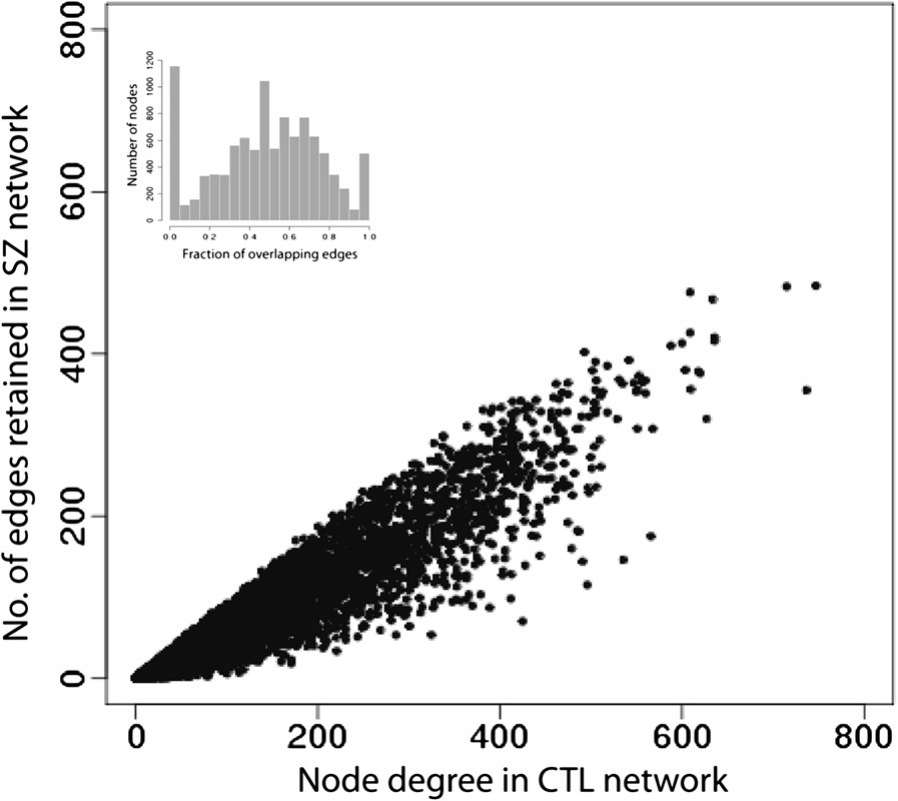
**Comparing node degree between networks.** To assess the node degree differences between networks, values of the node degree in the control network were plotted against the number of edges retained in the schizophrenia network. Each gene is represented by a dot in the plot, and all 12,582 genes were plotted. The presence of data points that deviate from the identity line indicate differences in gene-to-gene connections between the two networks. A histogram is also provided (inset) to illustrate that the distribution mean of the overlap is about fifty percent.

The small differences in global network properties observed between CTL and SZ suggests that there is an overall common coexpression structure of the prefrontal cortex. Fifty-seven percent of the edges (224,384 links) are the same in the two networks, much higher than expected by chance. The remaining 168,222 edges are not shared between the two networks (Figure 2). Subtle differences between the networks are also indicated by a higher maximum node degree in SZ (935) than CTL (737), and the increased number of non-connected nodes in CTL (2356) compared to SZ (2288). These differences could indicate subtle biological differences between the two networks, but are presumably at least partly due to the effect of noise.

**Figure 3:**
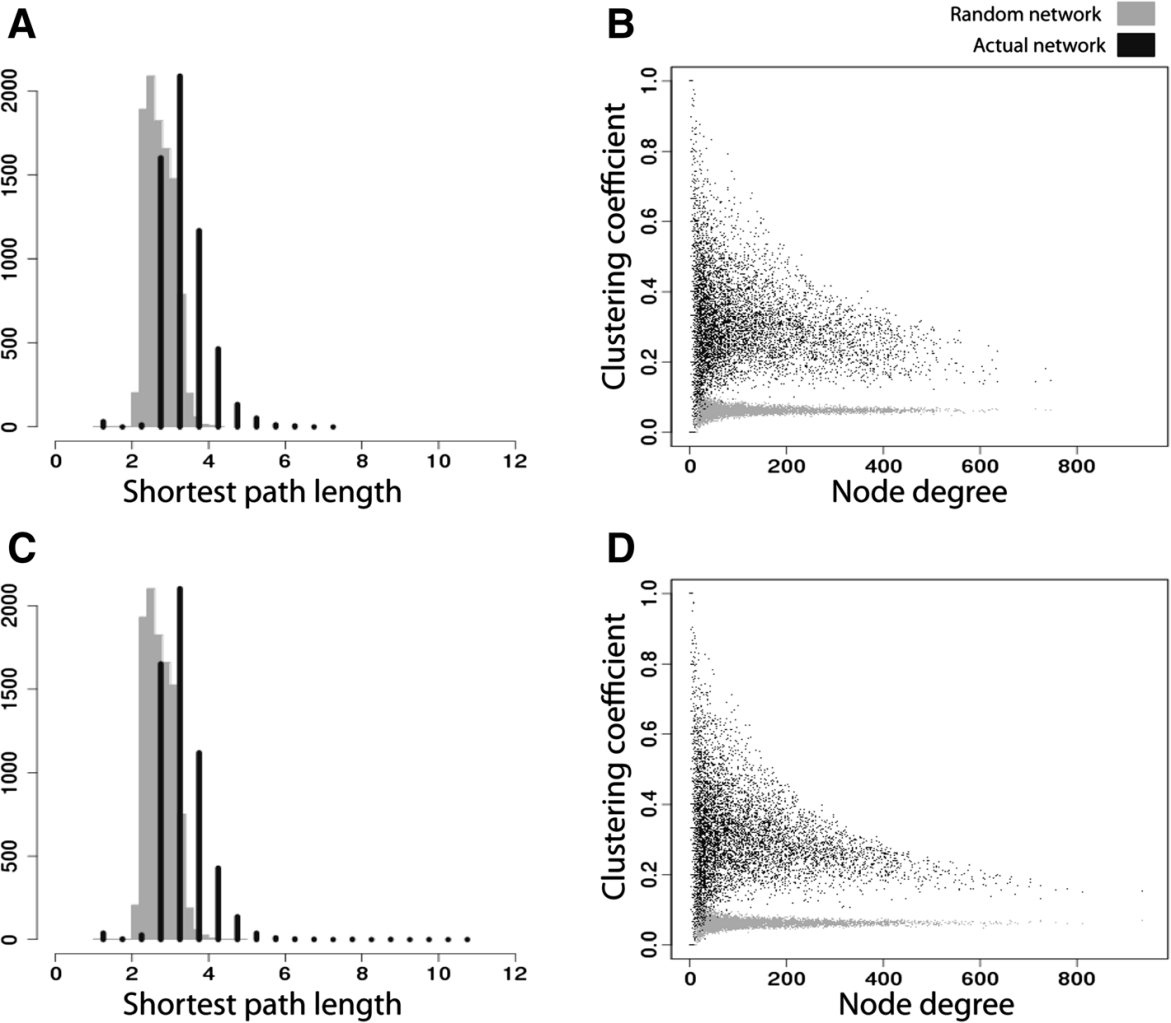
**Comparison to random network distributions.** For each network, we generated a corresponding random network by swapping edges and maintaining the same node degree distribution. **(A, C)** Shortest path length distribution of real networks are shifted slightly higher than corresponding random network distributions, but distributions between CTL and SZ are similar. Grey histograms reflect values from the random network, and black histograms represent the real network data. **(B, D)** Genes cluster into local communities with high number of interconnections compared to corresponding random networks. Black dots represent real network data and grey dots represent random network data.

In addition to comparing average network properties across the SZ and CTL networks to each other, we compared each separately to a node degree-matched random network (see Methods). For features based on connectivity (i.e. shortest path length and clustering coefficient), we found the observed distributions of both networks to be higher than compared to random networks. Shortest path length displayed slightly higher values than found in randomized networks (Figure 3A, C). Additionally, genes showed an increased clustering into local communities compared to genes from a randomized network with identical degree distribution (Figure 3B, D). Thus while, the SZ and CTL networks are similar, they are also clearly distinct from random networks with the same node degree distribution.

We next investigated network properties at the level of gene groups, focusing on our previously identified meta-signature of genes differentially expressed in schizophrenia [2]. The meta-signature of 94 genes (25 up-regulated and 69 down-regulated) will be referred to as SZUP and SZDOWN, respectively. Network properties were assessed for each gene set individually by taking an average across all genes in the group. These results are summarized in Table 3. For each gene set we computed the average values for shortest path length, cluster coefficient and node degree and evaluated differences observed between the control and schizophrenia networks. In general, both gene sets had a low mean node degree with respect to the network degree distribution of CTL and SZ, tending not to be ‘hubs’. For the SZUP gene set, we found higher node degree, shorter path length and an increased clustering coefficient in the SZ network, though these differences were not statistically significant (Wilcoxon- Rank Sum test p > 0.05). Conversely, the SZDOWN gene set exhibited a decreased node degree, larger path length and a lower clustering coefficient (Wilcoxon- Rank Sum test p = 0.05) in the SZ network. Thus, the properties of each gene set displayed an apparent trend between networks and those trends were observed to be opposite between the two gene sets.

**Table 3:** Schizophrenia gene set network properties

	**Up-regulated (25)**		**Down-regulated (69)**	
	**CTL**	**SZ**	**CTL**	**SZ**
**Node degree**				
Mean	63.9	83.5	127.4	106.2
Non-interacting nodes	2	2	7	3
**Edges** (within gene set)	12	23	129	144
**Shortest Path**				
Mean	3.28	3.14	3.31	3.48
*Random gene set comparison*				
Z-score	−0.58	−0.91	3.15	4.46
p-value	0.23	0.15	0	0
**Cluster Coefficient**				
Mean	0.35	0.38	0.32*	0.27*
*Random gene set comparison*				
Z-score	1.11	2.47	1.51	−0.607
p-value	0.14	0.005	0.06	0.28

*Difference is significant between CTL and SZ at p = 0.05.

The trends observed for SZUP and SZDOWN between the two networks were small and only marginally significant. To evaluate whether each of the individual gene set values were unusual in the networks we implemented three different methods of control. A first control was supplied by comparing observed network measures for SZUP and SZDOWN to a background distribution of 1000 randomly selected gene sets of matched size and node degree (see Methods). The difference between the observed values and background was assessed by computing z-scores and p-values, as reported in Table 3. Our strongest result was for the clustering coefficient of both gene sets. The p-values for the SZUP clustering coefficient indicate that the high value in SZ is significant when compared to a background distribution (p = 0.005). For SZDOWN, the high clustering coefficient in CTL showed a trend difference compared to the background distribution (p = 0.06). Together, these results converge to highlight that the gene neighbouring meta-signature gene sets are unusually highly coexpressed, with the SZUP genes displaying this property in the SZ network and the SZDOWN genes tending to display this in the CTL network.

**Figure 4:**
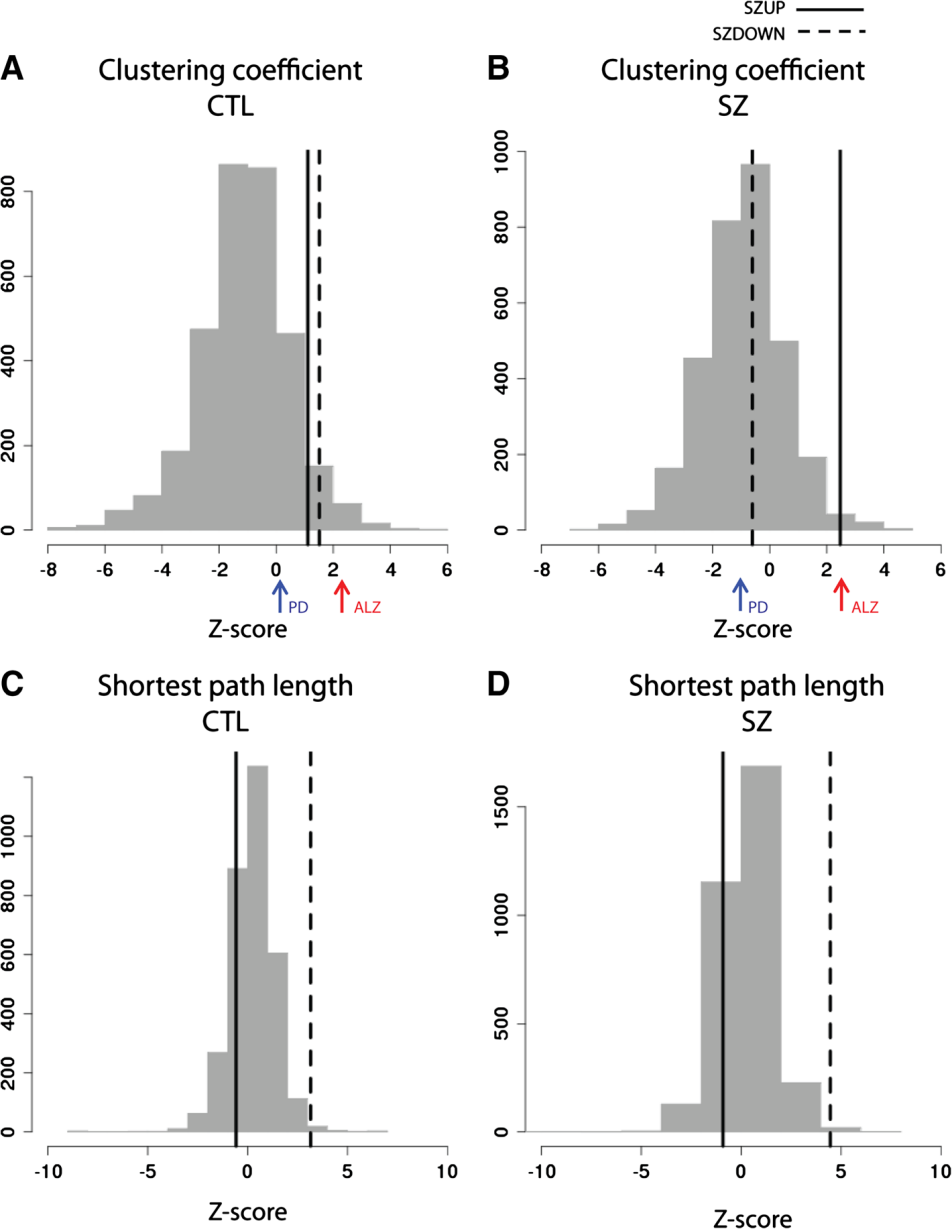
**Comparison of gene set properties to functional GO groups.** Histograms represent z-score distributions for cluster coefficient **(A, B)** and shortest path length **(C, D)** computed across 3,230 different GO groups in the control and schizophrenia networks. Z-scores represent the difference between the mean value of the network measure of the GO group compared to the mean of random gene sets of the same size and matched node degree. Dashed lines plotted represent the z-score obtained for the down-regulated meta-signature gene set and solid lines represent the z-score obtained for the up-regulated gene set. Z-score values for selected brain-related disease gene sets are displayed as coloured arrows on the x-axis. ALZ Alzheimer’s disease; PD Parkinson’s disease.

A second control was applied to examine whether or not the properties observed for SZUP and SZDOWN are a feature of other functionally grouped sets of genes. This is a more stringent control than simply comparing to random gene sets, because we are interested in properties of our genes that are unusual in schizophrenia compared to other functionally related groups of genes. We created 3,230 different functional gene sets based on GO terms and their associated genes. We assessed network measures for each functional gene set and compared the resulting z-scores to values for SZUP and SZDOWN (Figure 4). For the clustering coefficient, the z-score for SZUP is more distinguishable from GO group values in the SZ network compared to CTL. The opposite is true for the SZDOWN z-score values. Thus, the clustering coefficient is a unique property of our meta-signature genes not observed with other functional gene sets.

We next evaluated whether our meta-signature gene sets share network properties with gene sets associated with other brain-related disorders. We assembled gene sets for five different disorders, mostly based on findings from genetic association studies. For each disease gene set, z-scores were computed based on a background distribution and compared against the GO group z-scores in each network (Additional file 1: Table S1). Of particular interest are the results observed for clustering coefficient in the two networks. Interestingly, the Alzheimer’s disease gene group (Figure 4, red arrows) exhibited strikingly similar properties to SZUP in both networks despite having only one overlapping gene. Notably, the Parkinson’s disease gene group (Figure 4, blue arrows) follows a similar but more subtle trend as SZDOWN.

One concern is that these features are particular properties of the data sets we analyzed. In the absence of sufficient independent data, we assessed the robustness of the network measures observed for SZUP and SZDOWN using a jackknife procedure. In this process, we removed one of the seven datasets and regenerated aggregate CTL and SZ networks on the remaining six, for each study in turn. This yields seven pairs of jackknife networks. For each jackknifed network, the average shortest path length and clustering coefficient was computed for SZUP and SZDOWN and values were compared between networks (Additional file 2: Figure S1). For SZUP, we observed a general agreement of increasing clustering coefficient and consistently decreasing path length between CTL and SZ across all iterations. For SZDOWN, we found that only the clustering coefficient effects were robust to removing single data sets; the path length results proved to be more sensitive. Taken as a whole, these results are consistent with subtle network property differences for the SZUP and SZDOWN genes between the two networks.

**Figure 5:**
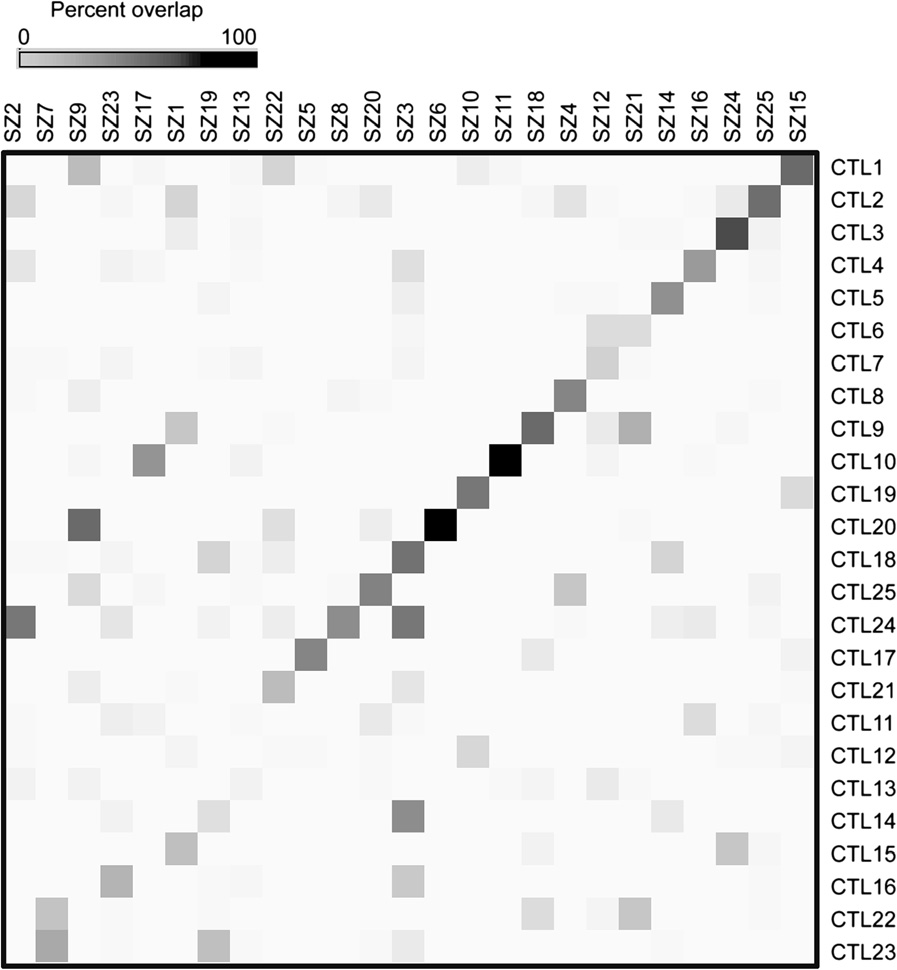
**Heatmap comparison of modules between networks.** Each network was clustered using WGCNA-based methods. Modules were compared between networks by computing the number of overlapping genes and a percent overlap was computed by taking the module of smaller size as the denominator. For each module pair the percent overlap is plotted with the scale provided.

Our analysis to this point examined either the entire networks or used supervised approaches to select sets of genes for analysis. We complemented this with an unsupervised method based on clustering [5]. This analysis was motivated in part by the observation that the meta-signature gene sets showed significant modularity differences between CTL and SZ. We hypothesized that there might be additional differences, beyond the parts of the network involving the meta-signature genes, or that this analysis might uncover additional features of the meta-signatures. Clustering resulted in 25 modules of varying sizes, in each network. An overlap comparison between the two sets of modules revealed strongly “matching” modules for 15 modules (p < 0.001, hypergeometric test; Figure 5), prompting further characterization of these modules according to disease association, cell-type specificity and functional roles.

We identified five modules in each network which displayed the most significant association with genes differentially expressed in schizophrenia (Wilcoxon rank-sum test; p < << 0.001), summarized in Table 4 (a list of all network modules is provided in Additional file 3: Table S2). In order to functionally characterize coexpression modules we cross-referenced the module gene lists with published lists of genes encoding markers of different CNS cell types (Table 5; Additional file 4: Table S3) and examined functional roles by GO enrichment (Additional file 5: Table S4 and Additional file 6: Table S5). The top schizophrenia-associated module in each network showed a significant enrichment of neuronal markers as well as genes belonging to GO categories related to the electron transport chain and oxidative phosphorylation (Module CTL24 and Module SZ3). A large proportion of the SZDOWN genes were also contained in this module, in both networks. Both networks also identified a module enriched for genes belonging to immune-related GO categories (Module CTL1 and Module SZ15). These modules were highly similar between networks, with a 71 percent overlap in gene membership and both associated with schizophrenia disease status. Cell type enrichment of this module in the control network identified 44 astrocyte marker genes (at low stringency threshold). Interestingly, the corresponding module in the schizophrenia network was reduced to eleven astrocyte marker genes. Also, we observed three SZUP genes (BAZ1A, TMEM176A, and FTL) in this module in the control network, but not in the schizophrenia network.

**Table 4:** Disease characterization of coexpression modules in each network

***Control Network***				
**Module Theme**	**Module No.**	**Module Size**	**SZUP Overlap**	**SZDOWN Overlap**
			**(25 genes)**	**(73 genes)**
Oxidative phosphorylation	CTL24	1996	0	34***
Glutamine metabolism	CTL20	504	7***	0
Myelination	CTL25	749	2	0
Immune response	CTL1	329	3**	0
Synaptic transmission	CTL18	291	0	10***
**Schizophrenia Network**				
**Module Theme**	**Module No.**	**Module Size**	**SZUP Overlap**	**SZDOWN Overlap**
			**(25 genes)**	**(73 genes)**
Oxidative phosphorylation	SZ3	1144	0	34***
Glutamine metabolism	SZ9	717	12***	1
Myelination	SZ20	711	2	0
Immune response	SZ15	198	0	0
Ubiquitination	SZ2	1424	0	12

The top five modules in each network were characterized by enrichment of genes that are differentially expressed in schizophrenia. Module themes were assigned based on Gene Ontology enrichment analysis. Disease effect p-values were computed by entering the t-statistic for the disease effect of each gene into a Wilcoxon rank-sum test by module. For all modules listed the disease effect p-values are p < << 0.001 and values can be found in the supplement. SZUP and SZDOWN are the up- and down-regulated schizophrenia gene sets previously identified in our meta-analysis [2]. **p < 0.01; ***p < < 0.001.

**Table 5:** Cell-type marker enrichment of coexpression modules in each network

***Control Network***				
		**Low stringency threshold ( > 4-fold)**		
**Module Theme**	**Module No.**	**Oligodendrocyte**	**Neuron**	**Astrocyte**
Oxidative phosphorylation	CTL24	43	253***	48
Glutamine metabolism	CTL20	23*	9	166***
Myelination	CTL25	115***	16	42
Immune-related	CTL1	7	10	44***
Synaptic transmission	CTL18	7	85***	9
**Schizophrenia Network**				
		**Low stringency threshold ( > 4-fold)**		
**Module Theme**	**Module No.**	**Oligodendrocyte**	**Neuron**	**Astrocyte**
Oxidative phosphorylation	SZ3	23	181***	22
Glutamine metabolism	SZ9	41***	20	203***
Myelination	SZ20	109***	16	28
Immune-related	SZ15	3	4	11
Ubiquitination	SZ2	34	157***	36

For the top disease-associated clusters in each network we report the number of genes that overlap with published lists of cell-type marker genes for oligodendrocytes , neurons, and astrocyte marker genes provided by [15]. Cell-type enrichment for all modules in each network and results for high stringency lists (> 10-fold) can be found in Additional file 4: Table 1. Hypergeometric probabilities were computed to evaluate significance of overlap. *p < 0.05; ***p < < 0.001.

The next two sets of modules that showed significant association with schizophrenia were also notable in high enrichment of cell-type marker genes. Module CTL25 and Module SZ20 were both significantly enriched for oligodendrocyte markers genes (e.g. OLIG2, MBP, MAG) with a 61 percent overlap in total gene membership, and each contained two SZUP genes. Highly ranked GO terms from functional enrichment analysis of each module included the presence of terms such as “ensheathment of neurons” and “regulation of action potential” suggesting this is was a myelination-related module. In contrast, modules CTL20 and SZ9 (which have 71 percent overlap in gene membership) were significantly enriched for astrocyte marker genes (Table 5; p < << 0.001). The common functional role suggested by GO enrichment centered around glutamine metabolism (e.g. GAD1, GAD2; see Additional file 5: Table S4 and Additional file 6: Table S5). SZUP genes also identified with this module, with a slightly higher number of genes identified in the SZ network (Table 3; p < < 0.001).

The final top disease-associated modules from each network, CTL18 and SZ2, were each associated with different cellular processes. Module CTL18 was enriched for neuronal marker genes and functional enrichment analysis revealed an association with genes related to neurotransmitter secretion. Furthermore, ten of the SZDOWN genes overlapped with this module. In the schizophrenia network, twelve SZDOWN genes (none of which overlap with the previous ten) were identified in Module SZ2. Module SZ2 is also neuron marker enriched but is associated with genes involved in ubiquitination. The two modules showed very little overlap with each other in terms of gene membership (with only six genes in common) and each highlighted different functional roles, yet both modules exhibited an association with schizophrenia in their respective networks.

We next evaluated the impact of covariates on our results by using expression changes known to be associated with age, brain pH and sex. Gene lists for these factors were compiled from a previous study of healthy control postmortem brain [16], and hypergeometric probabilities were computed to evaluate the significance of overlap with each module (Additional file 7: Table S6). No significant overlap was observed with the sex genes in either network. The age and pH genes displayed enrichment across the top modules which was mostly consistent between the control and schizophrenia networks. However, differential enrichment was observed for the immune response module, a trend similar to what we observed with SZUP genes and astrocyte marker genes. Expression data for the age and pH genes from the immune response module were plotted and fold change values were computed to examine the extent to which these genes might be affecting the network changes we observed (Additional file 8: Figure S2). We found expression to be variable within each cohort and differences in mean expression between cohorts were very small and not significant. Thus, it seems unlikely that there are large confounding effects of age and pH driving the changes we see in coexpression clustering between the two networks. We also addressed medication effects by cross-referencing lists of gene expression changes associated with lifetime antipsychotic use, to the top modules identified in our network analysis. The SMRI Online Genomics Database (https://www.stanleygenomics.org/) provides gene lists for several demographic variables which have been independently assessed using only the diseased subsets of each dataset. From the database we extracted significant gene lists (p < 0.001; FC > 1.2) pertaining to the effects of lifetime antipsychotics (69 genes) in subjects with schizophrenia. In the control network, a total of 34 medication-associated genes overlapped with our top modules, with the largest number found in the immune response module (Additional file 7: Table S6). In the schizophrenia network, roughly the same number of genes are re-distributed across the modules, but a significant overlap with the immune response module remains.

## Discussion

Our network-based approach for evaluating gene coexpression provides a novel assessment of coexpression patterns across seven large schizophrenia microarray datasets. We implemented a rank aggregation approach for network analysis revealing interesting patterns of molecular connectivity in the control and schizophrenia postmortem human brain. Overall, the two coexpression networks were very similar to one another. This is consistent with existing findings from network analysis in schizophrenia [8,10,11]. The control and schizophrenia networks shared a similar node degree distribution, and average values of path length and clustering coefficient taken across all nodes in the network were not significantly different. However, closer inspection revealed differences of potential biological significance.

To evaluate differences in gene-gene connectivity between networks, we initially focused on the network properties of 95 differentially expressed ‘schizophrenia genes’ as reported in our previous study of these same data sets [2]. This gene list was divided into two groups: 1) genes which are up-regulated in schizophrenia and 2) genes which are down-regulated in schizophrenia. We examined the network properties of each gene set within the control and schizophrenia networks and identified distinguishing features of our ‘schizophrenia genes’. The clustering coefficient, a measure which gives us insight into the community structure of nodes, proved to be an interesting characteristic of both gene sets. Importantly, we applied control protocols to demonstrate that this differential coexpression among the neighbourhood of ‘schizophrenia genes’ is not observed with other groups of “functionally related” genes and most other brain-related disease gene groups.

We also performed an assessment of modularity across all nodes in each network. Our results were comparable to two module coexpression analyses previously conducted in schizophrenia. The top disease-associated module in both networks was enriched for genes involved in oxidative phosphorylation and energy production This is consistent with results from Torkamani and colleagues [8] in which a combined network was generated from two expression datasets, both of which were used in our study. Moreover, our finding fits within a body of evidence suggesting mitochondrial dysfunction and defects in brain metabolism leading to oxidative stress in schizophrenia [17,18]. An oligodendrocyte marker enriched module was also identified in both CTL and SZ networks of our study and supported with the association of relevant GO terms related to myelination. In agreement, Roussos et al. [10] identified their top schizophrenia-associated module as highly enriched for oligodendrocyte marker genes as well as for genes associated with cytoskeleton rearrangement, axonal guidance and synaptogenesis. We note that the data used by Roussos et al. includes some samples contained in the Haroutunian dataset we used, albeit preprocessed in a different way (Table 6). The association of a myelination-related module with schizophrenia is in line with a wide range of reported white matter abnormalities linked to the illness [23] and genetic studies that have contributed a number of myelin and oligodendrocyte –related genes as candidate genes (e.g. APOD, PLP1, MAG) [24-26]. Torkamani and colleagues also identified an oligodendrocyte/myelin-related module in their network, but they did not observe any association with genes differentially expressed in schizophrenia.

**Table 6:** Datasets used in coexpression network analysis

**Dataset**	**Reference**	**Microarray Platform**	**Brain region(s)**	**No. of Subjects**
				**CTL:SZ**
Stanley Bahn	SMRI database	HG-U133A	Frontal BA46	31: 34
Stanley AltarC	SMRI database	HG-U133A	Frontal BA46/10	11: 9
*Mclean	HBTRC	HG-U133A	Prefrontal cortex (BA9)	26: 19
Mirnics	Garbett K. et al., 2008 [19]	HG-U133A/B	Prefrontal cortex (BA46)	6: 9
*Haroutunian	Katsel P. et al., 2005 [20]	HG-U133A/B	Frontal (BA10/46)	29: 31
GSE17612	Maycox P. et al., 2009 [21]	HG-U133 Plus 2.0	Anterior prefrontal cortex (BA10)	21: 26
*GSE21138	Narayan S. et al., 2008 [22]	HG-U133 Plus 2.0	Frontal (BA46)	29: 25

SMRI, Stanley Medical Research Institute; HBTRC, Harvard Brain Tissue Resource Centre (Mclean66 collection).

*Samples from these datasets have been used in previous coexpression network studies of schizophrenia [8,10].

**Figure 6:**
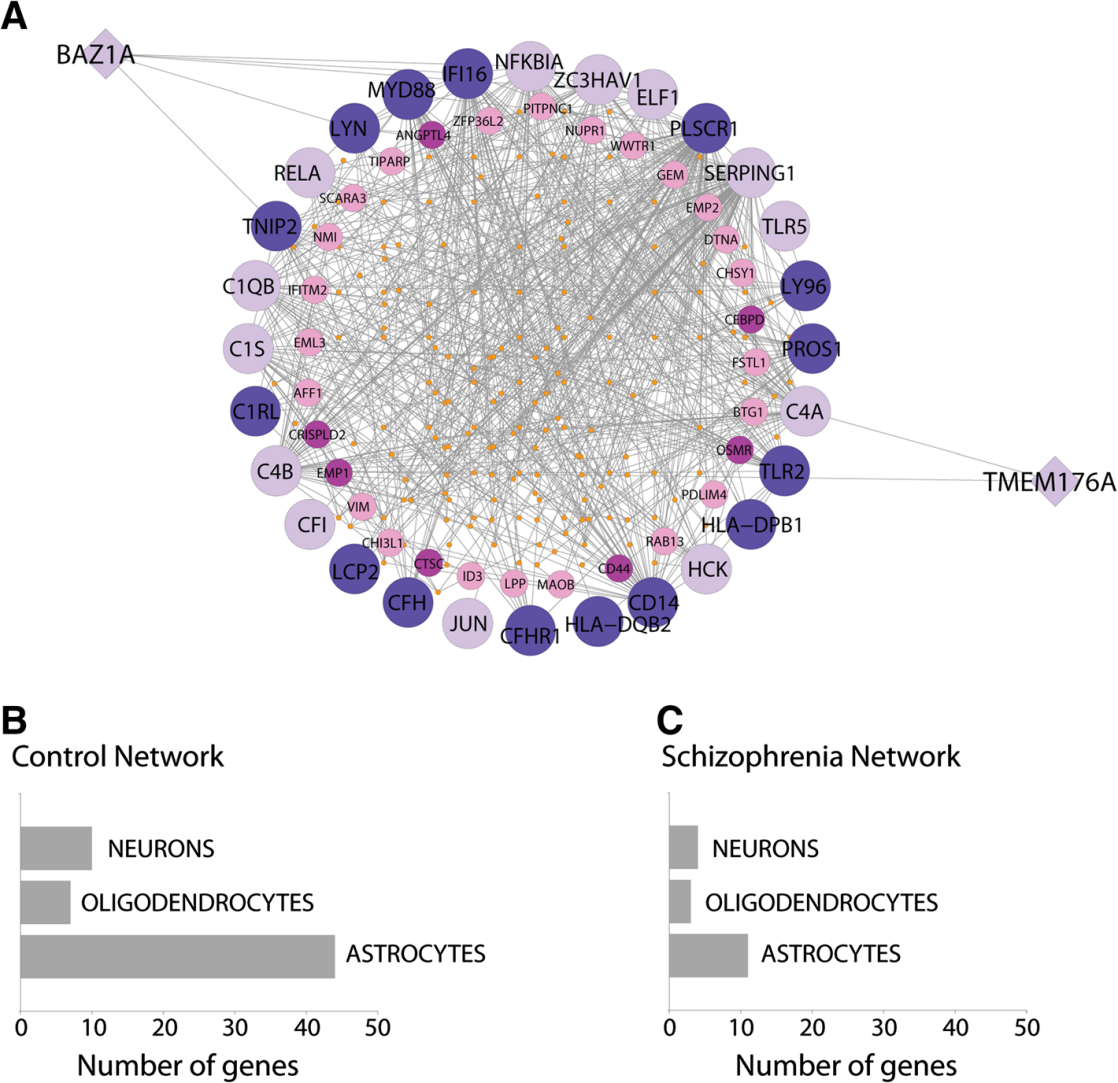
**Gene membership differences for the ‘immune’ module between networks. A)** Visualization of the ‘immune’ module in the control network is depicted by taking 28 immune activation genes (GO:002253) form the module and their associated connections within the module. This resulted in a sub-module of 229 nodes and 672 edges. Immune activation genes are represented by the larger nodes on the outer ring, astrocyte marker genes are represented by the smaller nodes on the inner ring and ‘schizophrenia genes’ are represented by diamond shaped nodes. Lighter colored nodes indicate the gene is not present in the schizophrenia network immune module while darker colored nodes indicate the gene is retained. **B)** and **C)** Bar graphs were used to illustrate the difference in cell-type marker membership for the ‘immune’ module in the two networks (using the low-stringency list).

In our analysis, an ‘immune’ module consistently appeared in both networks. While many genes in this module were conserved between the two networks, we found a number of differences suggesting alteration of immune-related processes in schizophrenia. In the control network, the immune response module was much larger in size and contained four times as many astrocyte marker genes (at low-stringency threshold) than in the schizophrenia network. A list of microglia marker genes were obtained from Bedard et al. (2007) [27] and also cross-referenced with top modules from each network. The numbers of overlapping genes with each module were few and did not change between the two networks. Although microglia are considered the resident macrophages of the brain providing the main arm of immune defense in the CNS, much evidence suggests that astrocytes also play an important role in the local regulation of immune reactivity [28]. In the control network immune module, 31 of the 44 overlapping astrocyte marker genes were coexpressed with one or more immune activation genes (Figure 6). Moreover, most of those astrocyte marker genes do not appear in the schizophrenia network module as a result of the loss of the immune activation genes. Torkamani et al. also identified an immune module, however, similar to the myelination module they failed to find any association with differentially expressed schizophrenia genes. Importantly, in our analysis three of the SZUP genes, specifically FTL, BAZ1A and TMEM176A, were identified in this module in CTL but not in SZ.

BAZ1A encodes a subunit of the chromatin assembly factor (ACF), which together with other proteins comprises the chromatin remodeling complex. Although it is not directly associated with the immune system, we found that it was coexpressed with a number of immune activation genes (Figure 6), suggesting a possible role in the transcriptional regulation of immune related genes. TMEM176A was also coexpressed with immune-related genes, complement component 4A (C4A) and complement component 4B (C4B), both of which are also not found in the SZ immune module (Figure 6). TMEM176A encodes a transmembrane protein, which together with TMEM176B when overexpressed has been shown to block dendritic cell maturation in rats [29]. Dendritic cells (DC) are immune cells found in most major organs, and their ability to regulate immunity is dependent on DC maturation. The brain has long been considered devoid of DC in the absence of inflammation, with microglia charged with many functional attributes commonly ascribed to DC. However, recent evidence has illustrated that DC are found in various tissue reservoirs within the steady-state CNS and are also potential players in brain immune surveillance [30].

Genes differentially expressed between schizophrenia subjects and healthy controls have also been identified through a recent combined analysis conducted on six of the same datasets used in our study [31]. Perez-Santiago et al. identified 117 up-regulated and 43 down-regulated probes of which 8 and 10 probes overlap with our up- and down- meta-signatures, respectively. The genes from Perez-Santiago et al. displayed a similar enrichment pattern as the Mistry et al. signature genes in the top modules in each of our networks. Specifically, the up-regulated genes showed a trend that coincides with trends observed with the astrocyte marker genes and SZUP genes. In the control network, the immune module contains 44 up-regulated genes and in the schizophrenia network the number of genes in the immune module is reduced to 19 genes. These findings provide additional support to our discussion on genes up-regulated in schizophrenia and alteration of immune-related processes.

The immunological link to the pathophysiology of schizophrenia suggested from our network analysis is not a new concept. Linkage and GWAS support an association of a broad section of markers in the major histocompatibility complex (MHC) region at 6p21.33 with schizophrenia [32,33] and many immune-related genes, genetic variants and haplotypes are also implicated in schizophrenia [34-36]. Several studies of gene expression in the postmortem PFC have reported alterations in immune and stress-response genes in subjects with schizophrenia [37-39]. Additionally, the investigation of gene expression in peripheral blood mononuclear cells (PBMCs) of drug-naïve schizophrenia subjects also supports alteration of immune-related processes [40-42]. Using our panel of schizophrenia genes found from meta-analysis we were able to identify unique features of the coexpression network and highlight relevant areas of dysfunction which may contribute to the pathophysiology of schizophrenia. However, our interpretation of the network model is based on GO enrichment and further investigation at the individual gene level will provide an explanation of higher resolution. We also sought evidence to support the plausibility of schizophrenia genetic risk variants driving the changes we observed. From the SZGene (http://www.szgene.org/) database we compiled the top 45 most reliable genetic associations based on findings from a meta-analysis [43] . This gene list was cross-referenced with the top modules from each network and we found no overlap with the immune module in either network.

One conclusion of our study is the effects on gene coexpression patterns in schizophrenia, like those on expression levels, are subtle in the face of other sources of variability. This suggests that while seven datasets is a much larger cohort than used in previous studies, our study would benefit from having additional data. Aggregating across a larger number of datasets has been shown to result in networks more comparable to PPI networks [44], and is likely to give more reliable interactions, but saturation of some properties was not reported to be achieved until >20 networks were combined. To help ameliorate concerns of robustness, we used a jackknife approach. The results from the jackknife analysis also address concerns regarding the reuse of datasets for both differential expression [2] and coexpression. We established that our findings are not overly sensitive to influence of any single data set. Also, for our null model comparisons we applied stringent controls whereby node degree was controlled for. Because node degree is one of the most important features of a network, it can drive numerous effects on other topological properties such as clustering coefficient and shortest path length. Thus controlling for node degree in generating null distributions is critical for avoiding false positives. Similarly, because we wished to identify network features which are associated with schizophrenia, we controlled for “generic” network features by comparing our schizophrenia gene sets to the properties of other “functionally coherent” gene groups, not just randomly selected genes. This approach was motivated by recent work from our group showing that generic multifunctionality effects can strongly skew the interpretation of gene networks [45].

As is the case with most postmortem brain studies in schizophrenia, our results should be interpreted in the context of several caveats. Samples used in this study were taken from patients having lived with schizophrenia for various lengths of time, and often having received medications. We cannot be sure that the changes we have identified are direct effects of the illness or are secondary to an underlying pathology. We were unable to obtain medication information for all samples used in this study (specifically, GSE17612 and Mclean66), and therefore were unable to precisely identify the extent to which antipsychotic use affects the results of our network analysis. We found that medication-associated genes overlap to a similar degree with the immune response module in both networks, which suggests that coexpression clustering patterns in schizophrenia are not driven by medication effects. The effects of other confounding variables (i.e. age, pH and sex) were also addressed in a similar manner, however we cannot exclude with certitude the possibility that the network properties we have identified are still in some way influenced by these extraneous factors. Also, samples used in our study comprise a heterogeneous collection of cell types from the DLPFC. While the majority of our datasets utilized samples from Brodmann areas 10 and 46, we also included one dataset from Brodmann area 9. Focusing on samples within a specific region would be ideal, as we avoid the potential dilution of cell-specific biological signals associated with schizophrenia. However, we included samples from all three Brodmann areas to maximize total sample size in our study and increase the power of our analysis.

Finally, we wish to stress limitations to the interpretation of coexpression networks. In contrast with other biological networks (i.e. protein interaction networks or metabolic networks) whose edges represent well-defined biological interactions, the edges in a coexpression network are a reduced representation of the correlation structure of the data. The edges are related to values of the pairwise correlation coefficient that are calculated from the expression data of the genes, and are dependent on the threshold applied to infer those networks. A connection between two genes in a coexpression network does not necessarily correspond with a connection in PPI networks, pathway or regulatory networks [46]. Thus, when studying gene coexpression networks it is important not to confuse the edges as direct physical interactions. Indeed, there is growing evidence that a substantial proportion of the variance in gene expression among replicate brain samples is explained by variance in cellular composition [5,6]. Our work supports this as we identify “modules” that are strongly associated with different cell type marker genes. Thus rather than identifying “physical interactions” among gene products, coexpression patterns to a large degree seem to reflect cell-type enriched expression, that is, expression in the same cell, but not necessarily finer levels of granularity such as a pathway. Changes in the composition of such modules might reflect changes in cellular states in subpopulations of cells, or changes in the associations of cell types with one another [47]. It is important not to interpret such differences as meaning that a physical interaction has been gained or lost among gene products.

## Conclusions

In summary we have contributed the largest meta-analysis of gene coexpression in schizophrenia. We evaluated various topological properties of the control and schizophrenia networks to reveal a shared coexpression structure between them. Characterization of functional clusters in each network with cell-type marker genes displayed differences that link together disease-related processes. Differentially expressed genes in schizophrenia also associate with biologically relevant clusters providing evidence for systems level dysfunction. Further research is required to disentangle these network findings to distinguish primary from secondary disease phenomena, but we hope our study will encourage new directions in the network biology of schizophrenia. Finally, our work demonstrates novel methodological approaches that can assist in ensuring that coexpression analyses yield biologically justifiable and robust results.

## Methods

### Data processing and quality control

Expression profiling data sets were selected on the basis of microarray platform, use of prefrontal cortex (BA 9, 10 or 46), the availability of information on covariates such as age, and finally the availability of the raw data [2]. Details on each of the seven datasets, including the source citation, can be found in Table 6. Data were preprocessed as described; briefly, expression levels were summarized, log_2_ transformed and normalized for each individual dataset using the R Bioconductor ‘affy’ package [48], with default settings for the RMA algorithm. Sample outliers were removed from each dataset based on an inter-sample correlation analysis, resulting in a total of 306 samples (153 from schizophrenia subjects, 153 from unaffected controls) across the seven data sets. Schizophrenia and control groups had no significant differences in age and PMI (Table 1). Sex differences were assessed by use of a chi-squared test for equality of proportion, and we observed no significant difference (p = 0.1). Brain pH was significantly different (*t*-test; p = 0.001). For each of the seven data sets, batch information was obtained using the ‘scan date’ stored in the CEL files; chips run on different days were considered different batches and batch effects for each dataset were removed using the ComBat algorithm [49].

### Gene coexpression networks

For each dataset, samples were separated into control and schizophrenia cohorts. Probes were mapped to genes using annotations provided in Gemma (http://www.chibi.ubc.ca/Gemma), which are based on stringent methods described in [50]. For genes mapping to multiple probes, the average expression value was retained. Only genes that were represented in all seven datasets were considered, leaving a total of 12,582 genes. This yielded seven expression data matrices for schizophrenia and seven for controls (one for each study). Separate networks were constructed for the schizophrenia and control groups based on previously described methods [44]. Briefly, a gene expression profile similarity matrix was computed for each cohort by taking the absolute value of the Pearson correlation between all possible gene pairs. Correlation values in the similarity matrix were replaced by ranks. These similarity matrices were aggregated by cohort across datasets by taking the mean rank for each gene pair. We previously showed that this aggregation procedure is a robust method for producing high-quality coexpression networks [44]. In keeping with previous work [44,51], the aggregated matrix was thresholded at 0.5% sparsity, resulting in an adjacency matrix of 392,606 connections for each of the control and schizophrenia cohorts.

### Random coexpression networks

To evaluate the significance of network measures across the whole network, formulation of appropriately randomized null models are required. We devised a procedure that results in a random network with the same number of genes and the same node degree distribution as the original data. Additionally, the node degree for each individual gene is preserved (i.e. each gene still has the same number of connections, but the specific genes to which it is connected to are scrambled). It has been previously shown that both PPI and coexpression networks show a correlation between node degree and gene multifunctionality [45]. Thus, by constraining each gene by its node degree we can systematically assess the significance of other topological properties of the network (i.e. clustering coefficient and shortest path length) while controlling for any potential multifunctionality bias in the microarray data. To create random networks, all gene pairs were assembled into an adjacency list (2 columns, 392,606 rows) and genes on one side of the edge were permuted. The resulting edges that created self-connections and/or duplicate gene pairs were isolated and permutation was re-applied to them. This was done iteratively until the number of conflicts was reduced to ten or less. These remaining conflicting edges were removed from the final random network.

### Network properties

We explored three different network properties, each of which is briefly described below.

#### Node degree

Each gene can be characterized by the number of connections it has, that is, the number of other genes it is significantly coexpressed with. This property is called the node degree. Node degrees were characterized by their distribution. For many biological networks the degree distribution has been characterized as ‘scale-free’, or at least ‘heavy tailed’. This can be observed by the quality of a linear fit of the distribution on log-log scale [52].

#### Shortest path

The shortest path length measures the shortest distance to get from one gene to another gene by traversing edges in the network. In an un-weighted network this is the least number of edges traversed to get between the two genes. We computed shortest paths using Dijkstra’s algorithm [53]. A value is obtained for a gene against every other gene in the network, and presented as the mean shortest path length across all genes. Genes without any direct neighbours are treated as missing values.

#### Clustering coefficient

The clustering coefficient of a gene indicates how connected the direct neighbours of a gene are to one another. It is the ratio of the number of connections in the neighbourhood of a node to the number of connections if the neighbourhood was fully connected. The clustering coefficient ranges from zero to one. A value of 1 would indicate that all the neighbours of a node are all connected to each other, or ‘cliquish’ in nature. A value of 0 would indicate that none of the neighbours of a node are connected to each other. This measure can only be computed for nodes that interact with more than one other node.

### Schizophrenia meta-signature network analysis

The meta-signature gene set of 25 up- and 73 down-regulated schizophrenia genes were obtained from the results of our meta-analysis of differential expression on the same datasets [2]. Four genes were removed from the down-regulated gene set as they were not present in the network, leaving a total of 94 ‘schizophrenia genes’. Throughout this chapter we will refer to these gene sets as SZUP and SZDOWN for the genes up- and down-regulated, respectively. Average values of shortest path length and clustering coefficient for the SZUP and SZDOWN gene sets were evaluated within each network. To estimate the relevance of the network measures for SZUP and SZDOWN, we implemented three important controls described below.

#### Random gene set comparison

For each meta-signature gene set, the average values of shortest path length and clustering coefficient were compared to a background distribution in each network. The background distribution was generated by randomly selecting 1000 gene sets. Random gene sets were constrained by size and node degree of the meta-signature gene set to control for multifunctional bias. To ensure a well-matched node degree for each random gene set, selection was done on a per-gene basis by choosing a random gene within ± 50 of its node degree rank. Z-scores were then computed to quantify the difference between the mean of the background distribution to the observed values for each network measure of SZUP and SZDOWN. For positive z-scores a p-value was computed reflecting how many random gene sets have values higher than the observed value. For negative z-scores a p-value was computed reflecting how many random gene sets have values less than the observed value.

#### Functional gene set comparison

Although our meta-signature of schizophrenia genes span a range of cellular functions, they possess a shared functional feature of altered expression in schizophrenia. Thus, it is important to assess whether the network properties we observe with our meta-signature gene sets are not just a property of gene groups that have lots of shared functional features. To control for this, we generated functionally characterized gene sets using the Gene Ontology (GO). From the GO database (http://www.geneontology.org/), we obtained 3,230 GO terms for which the associated gene set size ranged from 10–1000 genes. For each GO term we retrieved all human genes that were annotated with that term to compile a gene group for each GO term, also referred to as a functional gene set. Each of these functional gene sets were evaluated individually by comparison to a background distribution of randomly selected gene sets (of equivalent size and node degree), within each network. The distribution of z-scores obtained from 3,230 functional gene groups was plotted for each network and used to evaluate network properties of the meta-signature gene sets in reference to other functionally related gene sets.

#### Disease gene set comparison

To assess the network properties of our schizophrenia meta-signature genes in relation to other sets of disease-associated genes, we compiled disease gene lists for five different brain-related disorders. Gene sets were assembled for Alzheimer’s disease (http://www.alzgene.org/), Parkinson’s disease (http://www.pdgene.org/), multiple sclerosis (http://www.msgene.org/), and schizophrenia (http://www.szgene.org/) from their respective gene databases. Each database has been compiled based on findings from genetic association studies and provide gene lists on their website. The schizophrenia list obtained from SZGene (http://www.szgene.org/) comprised only the top 45 of the most reliable gene associations based on findings from a SZGene in-house meta-analysis. We also compiled an Autism spectrum disorder gene list from Toro et al. [54]. Average values of shortest path length and clustering coefficient were computed for all five disease gene sets. Network measures were compared to a background distribution of randomly selected gene sets and z-scores were compared against functional gene set z-score distributions.

### Network clustering

To extract modules (i.e. subset of nodes that are more densely connected to each other than to nodes outside the subset) from the control and schizophrenia networks we implemented a cluster-based algorithm based on methods described by [5]. Each adjacency matrix was transformed into a distance matrix by computing the topological overlap between all probe pairs [55]. Topological overlap measure (TOM) between two genes is calculated by comparing the direct connections of each. If two nodes connect to the same group of other nodes they are said to have ‘high topological overlap’. We used a generalization of this measure that enriches TOM’s sensitivity to longer ranging connections between nodes by incorporating the number of *m-*step neighbours (m = 2) that a pair of node share [55]. The TOM matrices were subjected to WGCNA-based methods [5], whereby hierarchical clustering was applied with average linkage, and the resulting tree was used to define network modules.

### Enrichment analysis

In order to determine which modules were associated with schizophrenia we looked for enrichment of differentially expressed genes found from our previous meta-analysis of the same data [2]. In addition to computing overlaps of SZUP and SZDOWN with each module, we also looked at enrichment by utilizing the t-statistic for the disease effect of each gene. T-statistics were entered into a Wilcoxon rank-sum test by module and resultant p-values were corrected for multiple testing using the Benjamini-Hochberg procedure.

To examine the functional roles encoded by these modules we used the Gene Ontology (GO) annotations and evaluated enrichment using the over-representation analysis (ORA) method in ErmineJ [44,56]. The ORA method evaluates the genes that meet a selection criterion to determine if there are gene sets (GO groups) which are statistically over-represented. The ORA method requires the entire list of genes and their associated scores and a score threshold must be selected. Binary scores were used to evaluate enrichment of each module; a value of 1 was assigned to genes with membership in the module and a value of 0 assigned to the rest of the genes. P-values for this method are computed by using the binomial approximation to the hypergeometric distribution, and then corrected for multiple testing using the Benjamini-Hochberg procedure. Modules were also evaluated for CNS cell type enrichment by cross-referencing the genes in each module with published lists of neuron, oligodendrocyte and astrocyte marker genes [15]. Cell type marker lists were compiled by extracting genes at a lower stringency threshold (as described in [8]) and a high stringency threshold (greater than 10-fold expression change). Hypergeometric probabilities were computed to evaluate the significance of overlap with cell type marker lists in each module.

## Abbreviations

CTL: Control network; SZ: Schizophrenia network; SZUP: Meta-signature genes up-regulated in schizophrenia; SZDOWN: Meta-signature genes down-regulated in schizophrenia; CNS: Central nervous system; PPI: Protein-protein interaction; GO: Gene ontology.

## Competing interests

The authors declare that they have no competing interests.

## Authors’ contributions

MM implemented algorithms and performed all of the analysis. JG constructed the rank aggregated coexpression networks and designed methods. PP oversaw design and execution of the study. PP and MM wrote the manuscript. All authors read and approved the final manuscript.

## Supplementary Material

Additional file 1: Table S1 Comparing brain-related disease gene set properties to functional GO groups. Click here for file

Additional file 2: Figure S1 Jackknifed network measures. For each jackknifed network (in which one dataset is removed), we computed shortest path length and clustering coefficient for SZUP and SZDOWN. To summarize trends observed in the jackknife analysis, we plotted clustering coefficient, shortest path length found in the CTL and SZ networks. Results from SZUP are found in A-B, and SZDOWN in C-D. Each line represents a different jackknifed network, with the legend indicating which dataset was removed. Click here for file

Additional file 3: Table S2 Disease characterization of all coexpression modules in each network. Modules in each network were characterized by enrichment of genes that are differentially expressed in schizophrenia. Disease effect p-values were computed by entering the t-statistic for the disease effect of each gene into a Wilcoxon rank-sum test by module. SZUP and SZDOWN are the up- and down-regulated schizophrenia gene sets previously identified in our meta-analysis [2]. Click here for file

Additional file 4: Table S3 Cell-type marker enrichment of coexpression modules in each network. For each cluster in the control and schizophrenia networks we report the number of genes that overlap with published lists of cell-type marker genes for oligodendrocytes , neurons, and astrocyte marker genes provided by [15]. Cell-type enrichment was computed for a low-stringency list (> 4-fold) and for a high stringency list (> 10-fold). Hypergeometric probabilities were computed to evaluate significance of overlap. Click here for file

Additional file 5: Table S4 Gene Ontology enrichment of top five disease modules in control. Click here for file

Additional file 6: Table S5 Gene Ontology enrichment of top five disease modules in schizophrenia networks, respectively. Click here for file

Additional file 7: Table S6 Enrichment of genes previously associated with other covariates. Click here for file

Additional file 8: Figure S2 Evaluating the effects of covariates on network modules. For the age up- and pH down regulated genes which are enriched in the immune response module of CTL, the expression data was plotted to evaluate differential expression between control and schizophrenia. A) Genes which remain in the SZ immune module; B) Genes that are lost from the SZ immune module. In either case, the expression for these genes is variable within each cohort and differences in mean expression between cohorts are very small and not significant. Click here for file
