# Supplementary material for: Meta-analysis of gene coexpression networks in the post-mortem prefrontal cortex of patients with schizophrenia and unaffected controls
Source: BMC Neurosci. 2013 Sep 26;14:105. doi: 10.1186/1471-2202-14-105 (PMC3849476; doi:10.1186/1471-2202-14-105)
Supplement: Additional file 1: Table S1 — Comparing brain-related disease gene set properties to functional GO groups. [file 1471-2202-14-105-S1.doc]

**Add3: Supplementary Table 1:** Comparing brain-related disease gene set properties to functional GO groups

|  | **Clustering Coefficient** | | **Shortest Path length** | |
| --- | --- | --- | --- | --- |
| **Disease gene set** | **CTL** | **SZ** | **CTL** | **SZ** |
| Alzheimer’s (ALZ) | 2.17 | 2.48 | 2.41 | 1.25 |
| Autism (ASD) | 0.65 | 0.13 | 0.73 | -1.23 |
| Multiple sclerosis (MS) | -1.18 | -0.95 | 1.44 | 0.38 |
| Parkinson’s disease (PD) | 0.06 | -0.98 | -0.70 | 1.08 |
| Schizophrenia SZGene (SCZ) | -0.17 | -1.02 | -0.60 | 0.65 |

Z-scores are reported for each disease gene set for network measures in the control and schizophrenia network. Z-scores represent the difference between the mean value of the network measure for the gene sets compared to random gene sets of the same size and matched node degree. These results were evaluated against findings reported in Figure 4 in the main text.
