# Supplementary figures and images for: Meta-analysis of gene coexpression networks in the post-mortem prefrontal cortex of patients with schizophrenia and unaffected controls
Source: BMC Neurosci. 2013 Sep 26;14:105. doi: 10.1186/1471-2202-14-105 (PMC3849476; doi:10.1186/1471-2202-14-105)

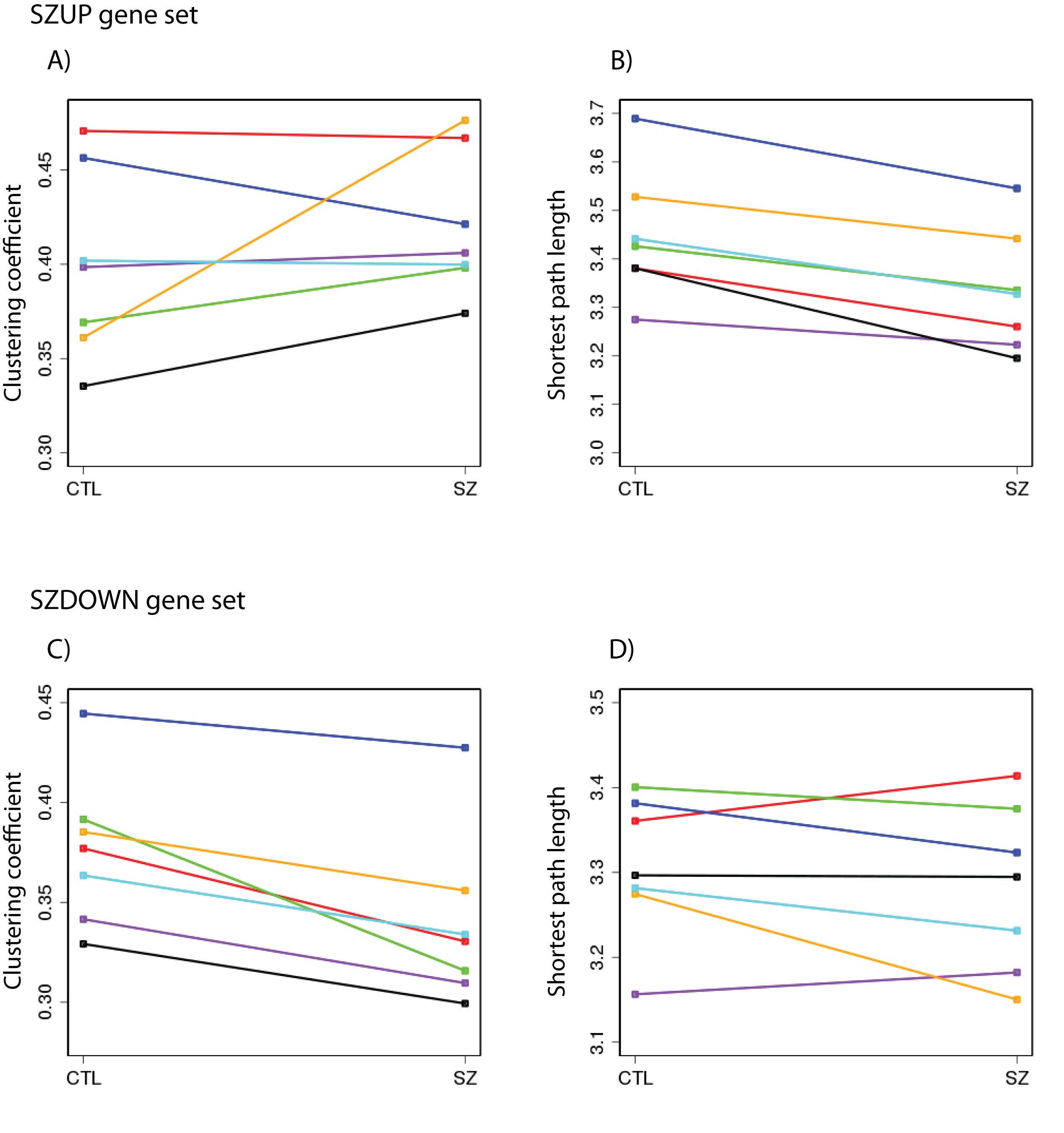

Supplement: Additional file 2: Figure S1 — Jackknifed network measures. For each jackknifed network (in which one dataset is removed), we computed shortest path length and clustering coefficient for SZUP and SZDOWN. To summarize trends observed in the jackknife analysis, we plotted clustering coefficient, shortest path length found in the CTL and SZ networks. Results from SZUP are found in A-B, and SZDOWN in C-D. Each line represents a different jackknifed network, with the legend indicating which dataset was removed. [file 1471-2202-14-105-S2.png]

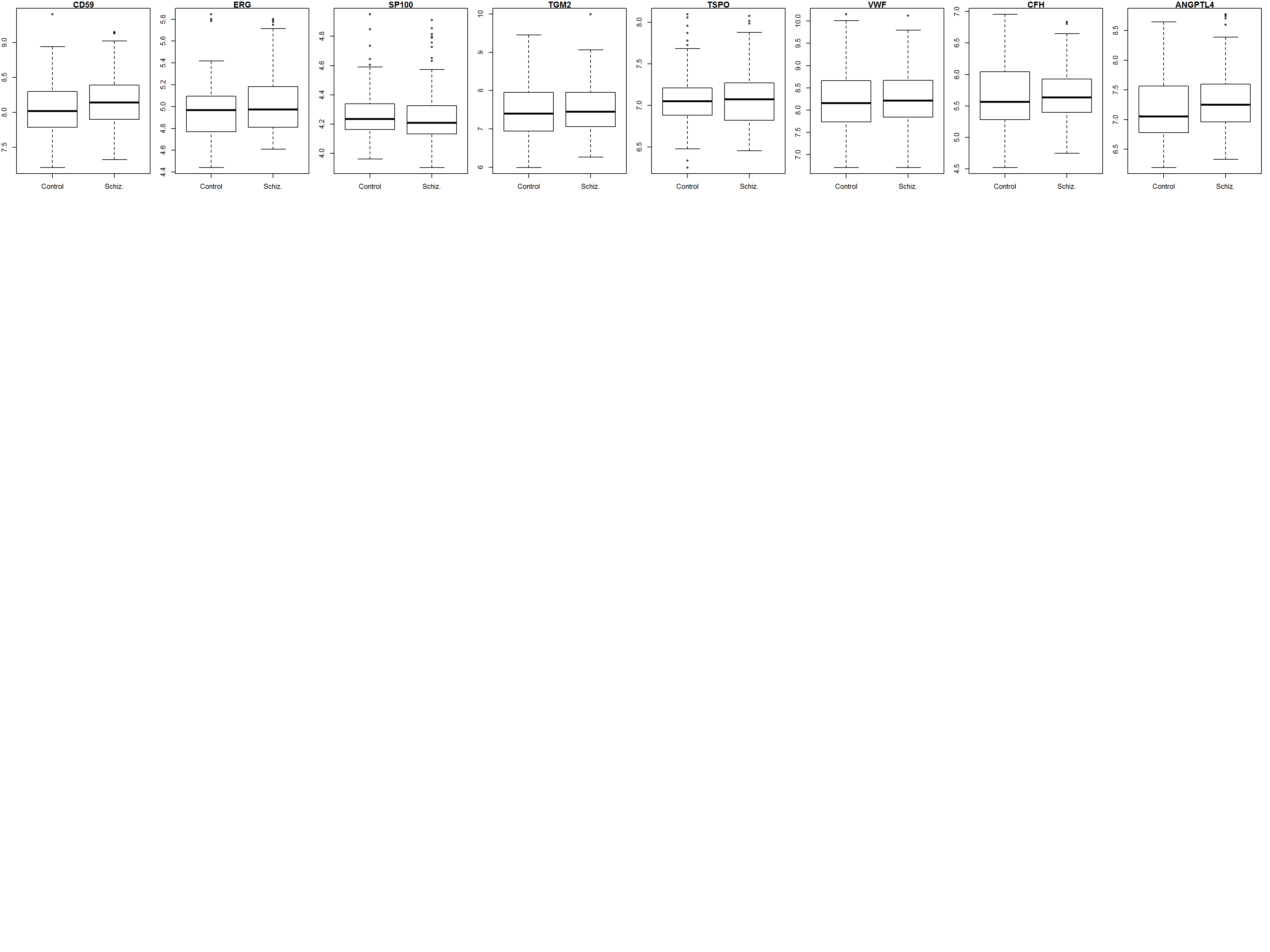

Supplement: Additional file 8: Figure S2 — Evaluating the effects of covariates on network modules. For the age up- and pH down regulated genes which are enriched in the immune response module of CTL, the expression data was plotted to evaluate differential expression between control and schizophrenia. A) Genes which remain in the SZ immune module; B) Genes that are lost from the SZ immune module. In either case, the expression for these genes is variable within each cohort and differences in mean expression between cohorts are very small and not significant. [file 1471-2202-14-105-S8.zip › add2/1864616886911546_add4.png]

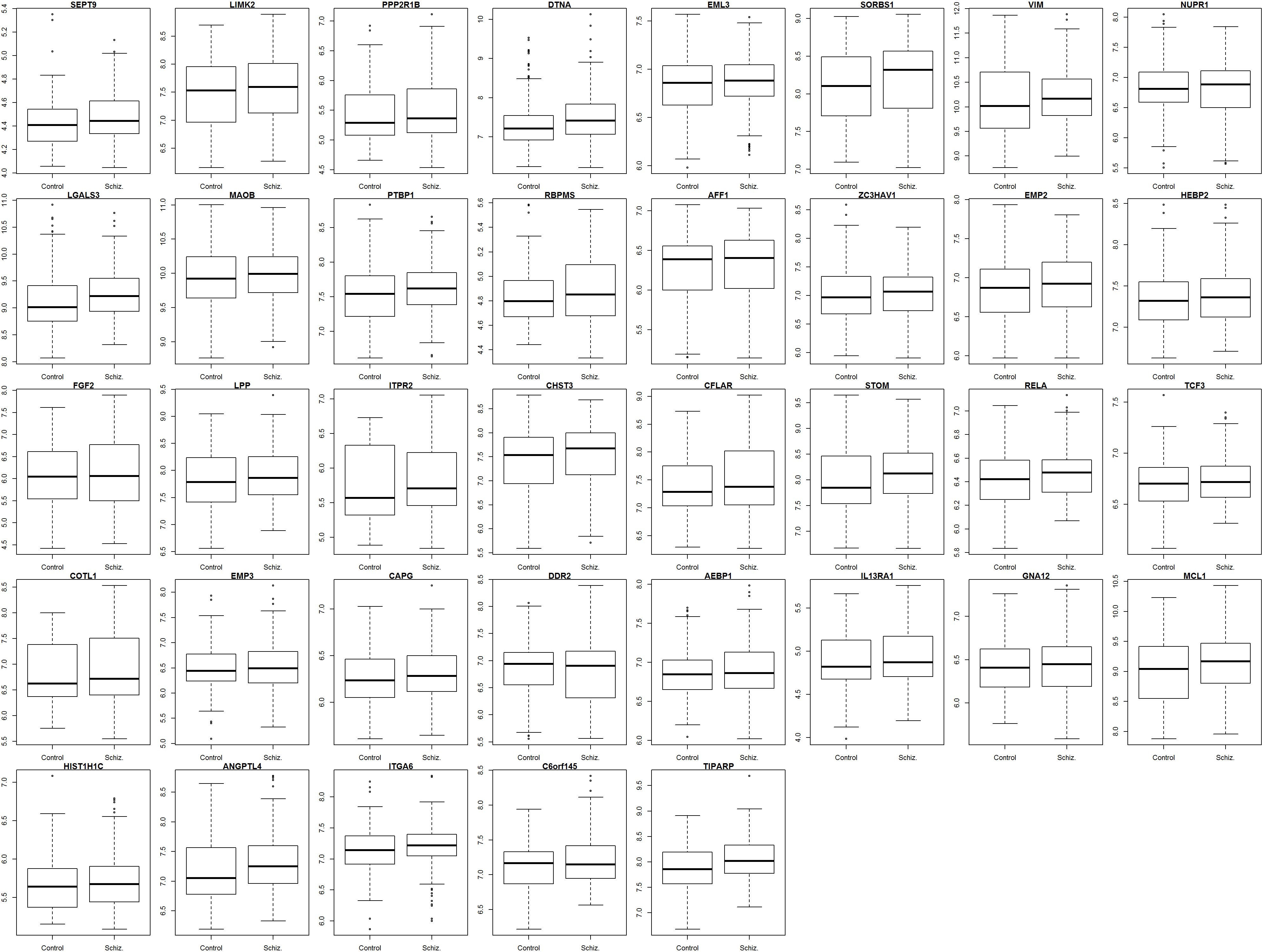

Supplement: Additional file 8: Figure S2 — Evaluating the effects of covariates on network modules. For the age up- and pH down regulated genes which are enriched in the immune response module of CTL, the expression data was plotted to evaluate differential expression between control and schizophrenia. A) Genes which remain in the SZ immune module; B) Genes that are lost from the SZ immune module. In either case, the expression for these genes is variable within each cohort and differences in mean expression between cohorts are very small and not significant. [file 1471-2202-14-105-S8.zip › add2/1864616886911546_add5.png]
