# Supplementary material for: Meta-analysis of gene coexpression networks in the post-mortem prefrontal cortex of patients with schizophrenia and unaffected controls
Source: BMC Neurosci. 2013 Sep 26;14:105. doi: 10.1186/1471-2202-14-105 (PMC3849476; doi:10.1186/1471-2202-14-105)
Supplement: Additional file 7: Table S6 — Enrichment of genes previously associated with other covariates. [file 1471-2202-14-105-S7.doc]

**Supplementary Table 6: Enrichment of genes previously associated with other covariates**

**Control Network**

| **Module Theme** | **Module No.** | **Module Size** | **Age Down**  **(1134 genes)** | **Age Up**  **(404 genes)** | **pH Down**  **(215 genes)** | **pH Up**  **(25 genes)** | **Medication**  **(69 genes)** |
| --- | --- | --- | --- | --- | --- | --- | --- |
| Oxidative phosphorylation | CTL24 | 1996 | 498*** | 4 | 10 | 17*** | 4 |
| Glutamine metabolism | CTL20 | 504 | 12 | 90*** | 83*** | 0 | 4 |
| Myelination | CTL25 | 749 | 10 | 57*** | 29*** | 0 | 5 |
| Immune response | CTL1 | 329 | 3 | 38*** | 17*** | 0 | 21*** |
| Synaptic transmission | CTL18 | 291 | 118*** | 2 | 1 | 2 | 0 |

**Schizophrenia Network**

| **Module Theme** | **Module No.** | **Module Size** | **Age Down**  **(1134 genes)** | **Age Up**  **(404 genes)** | **pH Down**  **(215 genes)** | **pH Up**  **(25 genes)** | **Medication**  **(69 genes)** |
| --- | --- | --- | --- | --- | --- | --- | --- |
| Oxidative phosphorylation | SZ3 | 1144 | 364*** | 2 | 4 | 13*** | 1 |
| Glutamine metabolism | SZ9 | 717 | 17 | 127*** | 100** | 1 | 15*** |
| Myelination | SZ20 | 711 | 15 | 47*** | 18 | 0 | 1 |
| Immune response | SZ15 | 198 | 3 | 9 | 2 | 0 | 12*** |
| Ubiquitination | SZ2 | 1424 | 273*** | 3 | 2 | 7** | 1 |

The top five disease-associated modules in each network were assessed for enrichment of genes associated with confounding variables. Gene lists for age and pH were compiled from a previous study of healthy control postmortem brain (as described in Mistry and Pavlidis, 2010), Medication gene lists were obtained from The SMRI Online Genomics Database (<https://www.stanleygenomics.org/>) . Hypergeometric probabilities were computed to evaluate significance of overlap. *p < 0.05; **p < 0.01; ***p << 0.001.
